# Journal of NeuroEngineering and Rehabilitation reviewer acknowledgement 2014

**DOI:** 10.1186/s12984-015-0008-6

**Published:** 2015-02-24

**Authors:** David J Reinkensmeyer

**Affiliations:** Department of Mechanical and Aerospace Engineering, University of California, Irvine, USA; Department of Anatomy and Neurobiology, University of California, Irvine, USA; Department of Biomedical Engineering, University of California, Irvine, USA; Department of Physical Medicine and Rehabilitation, University of California, Irvine, USA

## Abstract

The editors of *Journal of NeuroEngineering and Rehabilitation* would like to thank all of our reviewers who have contributed to the journal in volume 11 (2014).

**Daijiro Abe**

Japan

**U. Rajendra Acharya**

Singapore

**Diane E. Adamo**

United States of America

**Sergei Adamovich**

United States of America

**Allan Adkin**

Canada

**Valentina Agostini**

Italy

**Zaghloul Ahmed**

United States of America

**Mark Albert**

United States of America

**Joan F. Alonso**

Spain

**Farshid Amirabdollahian**

United Kingdom

**Philippe Archambault**

Canada

**J. Hans Arendzen**

Netherlands

**John Arkwright**

Australia

**Panagiotis Artemiadis**

United States of America

**Alexander Aruin**

United States of America

**Oskar Aszmann**

Austria

**Musa Audu**

United States of America

**Louis Awad**

United States of America

**Ali Bahramisharif**

Netherlands

**Alberto Baldini**

Italy

**Subhasis Banerji**

Singapore

**Robert John Barry**

Australia

**Dorothy Barthélemy**

Canada

**Gabor Barton**

United Kingdom

**Rezaul Begg**

Australia

**Nikunj Bhagat**

United States of America

**Tanvi Bhatt**

United States of America

**Daniele Bibbo**

Italy

**Sandra Billinger**

United States of America

**Martin Bilodeau**

Canada

**Stuart Binder-Macleod**

United States of America

**Andreanne Blanchette**

Canada

**Amy Blank**

United States of America

**Florian Bodranghien**

Belgium

**Richard Bohannon**

United States of America

**Matthieu Boisgontier**

Belgium

**Frank Borg**

Finland

**Jörgen Borg**

Sweden

**Dennis Bourbeau**

United States of America

**Mark Bowden**

United States of America

**John-Stuart Brittain**

United Kingdom

**Cynthia Brown**

United States of America

**Dustin Bruening**

United States of America

**Michelle Burack**

United States of America

**Etienne Burdet**

United Kingdom

**Monica Cameirao**

Portugal

**Filippo Camerota**

Italy

**Leonardo Cappello**

Italy

**Stephanie Carey**

United States of America

**Tom Carlson**

United Kingdom

**Stephen Carp**

United States of America

**Helder Carvalho**

Portugal

**Margherita Castronovo**

Germany

**Brian Caulfield**

Ireland

**Massimo Cenciarini**

Germany

**Rosa H. M. Chan**

Hong Kong

**Shuo-Hsiu Chang**

United States of America

**Yao-Jen Chang**

Taiwan

**Xiang Chen**

China

**Félix Chénier**

Canada

**Carmelo Chisari**

Italy

**Veronica Cimolin**

Italy

**Pietro Cipresso**

Italy

**Edward (Ted) Clancy**

United States of America

**Ross Clark**

Australia

**Sue Cobb**

United Kingdom

**Steve Collins**

United States of America

**Silvia Conforto**

Italy

**Elaine Corbett**

Australia

**Patrick Costigan**

Canada

**Elaine Coulter**

United Kingdom

**Patrick Crago**

United States of America

**Frederic Crevecoeur**

Canada

**Dustin Crouch**

United States of America

**Xingran Cui**

United States of America

**Armin Curt**

Switzerland

**Marco D'Alonzo**

Italy

**Carmen D'Anna**

Italy

**Paulo de Freitas**

Brazil

**Ray de Leon**

United States of America

**Ana de los Reyes Guzmán**

Spain

**Helios de Rosario**

Spain

**Jesse Dean**

United States of America

**Philippe Dedieu**

France

**Ugo Della Croce**

Italy

**Thibault Deschamps**

France

**Nandini Deshpande**

Canada

**Judith Deutsch**

United States of America

**Michael Devlin**

Canada

**Nuno Dias**

Portugal

**Dan Ding**

United States of America

**An Do**

United States of America

**Christian Dohle**

Germany

**Takehiko Doi**

Japan

**Strahinja Dosen**

Germany

**Jennifer Dunn**

New Zealand

**Lucy Dunne**

United States of America

**Assaf Dvorkin**

United States of America

**Gerold Ebenbichler**

Austria

**Gail Eskes**

Canada

**Debbie Espy**

United States of America

**Alberto Esquenazi**

United States of America

**Alfonso Fasano**

Canada

**Philippe Faure**

France

**Xin Feng**

United States of America

**Jing Feng**

United States of America

**Simona Ferrante**

Italy

**Giancarlo Ferrigno**

Italy

**Daniel Ferris**

United States of America

**Peter Feys**

Belgium

**Fanny Ficuciello**

Italy

**Hillel Finestone**

Canada

**James Finley**

United States of America

**Lee Fisher**

United States of America

**Kevin Fite**

United States of America

**Bertine Fleerkotte**

Netherlands

**Robert Flint**

United States of America

**Gerard Fluet**

United States of America

**Stephen Foldes**

United States of America

**Kenneth Fong**

Hong Kong

**Emily Fox**

United States of America

**Jason Friedman**

Israel

**Gail Frost**

Canada

**David Gabriel**

Canada

**Alejandro Galan-Mercant**

Spain

**Sujay Galen**

United States of America

**Philippe Gallien**

France

**Brook Galna**

United Kingdom

**Fan Gao**

United States of America

**Nicolas Garcia Aracil**

Spain

**Laura Gastaldi**

Italy

**Lynne Gauthier**

United States of America

**Xinling Geng**

China

**Alireza Gharabaghi**

Germany

**Giuseppina Gini**

Italy

**Leonardo Gizzi**

Germany

**Manuel González-Sánchez**

Spain

**Keith Gordon**

United States of America

**Alena Grabowski**

United States of America

**Robert Gregg**

United States of America

**Philip Grewe**

Germany

**Michael Grey**

United Kingdom

**Giuliana Grimaldi**

Belgium

**Eleni Grimpampi**

Italy

**Emmanuel Guigon**

France

**Etienne Guillaud**

France

**Lan-Yuen Guo**

Taiwan

**Michael Hahn**

United States of America

**Courtney Hall**

United States of America

**Gyu Cheol Han**

South Korea

**Noor Hanif**

United Kingdom

**Andrew Hansen**

United States of America

**Orla Hardiman**

Ireland

**Levi Hargrove**

United States of America

**Chris Hass**

United States of America

**Christopher Hasson**

United States of America

**Anna Hatton**

Australia

**Malcolm Hawken**

United Kingdom

**Daniel Heitzmann**

Germany

**Trent Herda**

United States of America

**Stefan Hesse**

Germany

**Shivayogi Hiremath**

United States of America

**Ales Holobar**

Slovenia

**Tiago Hori**

Canada

**Elizabeth Hsiao-Wecksler**

United States of America

**Ru-Lan Hsieh**

Taiwan

**Jwu-Sheng Hu**

Taiwan

**Yong Hu**

Hong Kong

**Hsiu-Chen Huang**

Taiwan

**He (Helen) Huang**

United States of America

**Christopher Hurt**

United States of America

**Ing-Shiou Hwang**

Taiwan

**Trienke IJmker**

Netherlands

**Marco Iosa**

Italy

**Md. Anamul Islam**

Malaysia

**Mark Ison**

United States of America

**Nathanael Jarrassé**

France

**Steve Jax**

United States of America

**Arun Jayaraman**

United States of America

**Jean-Yves Jenny**

Fiji

**Winnie Jensen**

Denmark

**Ning Jiang**

Germany

**Reva Johnson**

United States of America

**Alon Kalron**

Israel

**Derek Kamper**

United States of America

**Guiyeom Kang**

Ireland

**Shigehisa Kawakami**

Japan

**Stephen Kelly**

United Kingdom

**Trisha Kesar**

United States of America

**Rami Khushaba**

Australia

**Kevin Kilgore**

United States of America

**Gyoung-Mo Kim**

South Korea

**Sohee Kim**

South Korea

**Kristof Kipp**

United States of America

**Rachel Kizony**

Israel

**Verena Klamroth-Marganska**

Switzerland

**Bert Kleine**

Netherlands

**Rudolf Klemetti**

Finland

**Marco Knaflitz**

Italy

**Maria Knikou**

United States of America

**Toshiki Kobayashi**

United States of America

**Silvia Erika Kober**

Austria

**Dylan Kobsar**

Canada

**Rodger Kram**

United States of America

**Sharon Kramer**

Australia

**Hermano Igo Krebs**

United States of America

**Carmen Krewer**

Germany

**Hollis Krug**

United States of America

**Todd Kuiken**

United States of America

**Dinesh Kumar**

Australia

**Jason Kutch**

United States of America

**Laurel Kuxhaus**

United States of America

**Ruben Kuzniecky**

United States of America

**Peter Kyberd**

Canada

**Joris Lambrecht**

United States of America

**Claudine Lamoth**

Netherlands

**Beom Chan Lee**

United States of America

**Hyunglae Lee**

United States of America

**Sang Wook Lee**

United States of America

**Yun-Ju Lee**

United States of America

**Yafi Levanon**

Israel

**Mindy Levin**

Canada

**Beth Lewandowski**

United States of America

**Sandra Lewis**

United Kingdom

**Jan Lexell**

Sweden

**Guanglin Li**

China

**Karen Li**

Canada

**Ke Li**

China

**Li Li**

United States of America

**Meiling Li**

China

**Qingguo Li**

Canada

**Joachim Liepert**

Germany

**Fang Lin**

United States of America

**Jonathan Lin**

Canada

**Suh-Jen Lin**

United States of America

**Pavel Lindberg**

France

**Simon Little**

United Kingdom

**Beth Lopour**

United States of America

**Stephen Lord**

Australia

**Richard Lovering**

United States of America

**Madeleine Lowery**

Ireland

**Marilyn MacKay-Lyons**

Canada

**Andreas Maier**

Germany

**Margaret Mak**

Hong Kong

**Philippe Malcolm**

Belgium

**Martina Mancini**

United States of America

**Brad Manor**

United States of America

**Mario Manto**

Belgium

**Marin Manuel**

France

**Robin Marcus**

United States of America

**Susan Marion**

United States of America

**Alain Martin**

France

**Joel Martin**

United States of America

**Roshan Joy Martis**

Singapore

**Sarah Mason**

United Kingdom

**Giulia Matrone**

Italy

**Alessandro Mauro**

Italy

**Claudia Mazzà**

Italy

**Suzanne McDonough**

United Kingdom

**Bradford McFadyen**

Canada

**Paul McGeoch**

United States of America

**Kevin McGill**

United States of America

**Linda McLean**

Canada

**Ranjana Mehta**

United States of America

**Sabato Mellone**

Italy

**William Memberg**

United States of America

**Rochelle Mendonca**

United States of America

**Thomas Mergner**

Germany

**Anat Mirelman**

Israel

**Sambit Mohapatra**

United States of America

**Filippo Molinari**

Italy

**Marco Molinari**

Italy

**Luis Montesano**

Spain

**J. Leon Morales-Quezada**

United States of America

**Juan C. Moreno**

Spain

**Steven Morrison**

United States of America

**Winfred Mugge**

Netherlands

**Gernot Müller-Putz**

Austria

**Sandro Mussa-Ivaldi**

United States of America

**Kristin Musselman**

Canada

**Pratik Mutha**

India

**Jiro Nakano**

Japan

**Dominic Nathan**

United States of America

**Tobias Nef**

Switzerland

**Francesco Negro**

Germany

**Elizabeth Nightingale**

Australia

**Domen Novak**

Switzerland

**Toru Ogata**

Japan

**Shinya Ogaya**

Japan

**Lauro Ojeda**

United States of America

**Taher Omari**

Australia

**Kenneth Ottenbacher**

United States of America

**Kerstin Palombaro**

United States of America

**Enrica Papi**

United Kingdom

**Matthew Parkes**

United Kingdom

**Ilaria Pasciuto**

Italy

**Ben Patritti**

Australia

**Carolynn Patten**

United States of America

**Kara Patterson**

Canada

**Lorna Paul**

United Kingdom

**Alessandra Pedrocchi**

Italy

**Joel Perry**

Spain

**Angel Peterchev**

United States of America

**Mark Peterson**

United States of America

**Daniel Peterson**

United States of America

**Gert Pfurtscheller**

Austria

**Elena Pirogova**

Australia

**Mark Pitkin**

United States of America

**Dejan Popovic**

Serbia

**Mira Popovic**

Serbia

**Sigal Portnoy**

Israel

**Martin Posch**

Austria

**Gerdienke B. Prange**

Netherlands

**Rachel Proffitt**

United States of America

**Alessio Pulizzotto**

Italy

**Kai Qian**

United States of America

**Robrecht Raedt**

Belgium

**Danny Rafferty**

United Kingdom

**Fabian Rast**

Switzerland

**Rafael Raya**

Spain

**Jeffrey Reinbolt**

United States of America

**Olivier Remy-Neris**

France

**Daniel Renjewski**

Switzerland

**Christopher Rhea**

United States of America

**Sarah Ridge**

United States of America

**Laura Rocchi**

Italy

**Eduardo Rocon**

Spain

**Eric Rombokas**

United States of America

**Noah Rosenblatt**

United States of America

**Diana Ruiz Bueno**

Spain

**Ruediger Rupp**

Germany

**David Russ**

United States of America

**Manning Sabatier**

United States of America

**Hooshang Saberi**

Iran

**Brian Sandroff**

United States of America

**Amirehsan Sarabadani Tafreshi**

Switzerland

**Makoto Sasaki**

Japan

**Katherine Saul**

United States of America

**Zimi Sawacha**

Italy

**Lumy Sawaki**

United States of America

**Andrew Sawers**

United States of America

**Gregory Sawicki**

United States of America

**Edward Sazonov**

United States of America

**Sydney Schaefer**

United States of America

**Philip Schatz**

United States of America

**Erik Scheme**

Canada

**Sheila Schindler-Ivens**

United States of America

**Maurizio Schmid**

Italy

**Peter Schneider**

Germany

**Alfred Schouten**

Netherlands

**Stylianos Scordilis**

United States of America

**Stephen Scott**

Canada

**Thomas Seel**

Germany

**Ava Segal**

United States of America

**Ervin Sejdic**

United States of America

**Masaki Sekine**

Japan

**Jon Sensinger**

Canada

**Na Jin Seo**

United States of America

**Giacomo Severini**

United States of America

**Ludy Shih**

United States of America

**Pete Shull**

China

**Kathryn Sibley**

Canada

**Ann Simon**

United States of America

**Mikhail Simonov**

Italy

**Tarkeshwar Singh**

United States of America

**Sue Ann Sisto**

United States of America

**Manoj Sivan**

United Kingdom

**Lauren Smith**

United States of America

**Marco Soares dos Santos**

Portugal

**Jacob Sosnoff**

United States of America

**Patrick Sparto**

United States of America

**Shmuel Springer**

Israel

**Daniel Stashuk**

Canada

**Despina Stavrinos**

United States of America

**Joel Stein**

United States of America

**Jill Stewart**

United States of America

**Ian Stokes**

United States of America

**Oliver Stoller**

Switzerland

**Jake Streepey**

United States of America

**Fong-Chin Su**

Taiwan

**Sandeep Subramanian**

Canada

**Tomohiko Takei**

Japan

**Andrew Tan**

United States of America

**Nitish Thakor**

United States of America

**Michael Thaut**

United States of America

**Alessandro Tognetti**

Italy

**Craig Tokuno**

Canada

**Diego Torricelli**

Spain

**Yannick Tousignant-Laflamme**

Canada

**Diana Trojaniello**

Italy

**Randy Trumbower**

United States of America

**Inna Tsirlin**

Canada

**Carole Tucker**

United States of America

**Andrea Turolla**

Italy

**Ailie Turton**

United Kingdom

**Edwin van Asseldonk**

Netherlands

**Herman van der Kooij**

Netherlands

**Bram van Dun**

Australia

**Richard van Emmerik**

United States of America

**Natalie Vanicek**

United Kingdom

**Giovanni Vecchiato**

Italy

**Beatrix Vereijken**

Norway

**Paul F.M.J. Verschure**

Spain

**Bruce Volpe**

United States of America

**Tim Wagner**

United States of America

**Erik Walbeehm**

Netherlands

**Conrad Wall**

United States of America

**Jinsung Wang**

United States of America

**Yijun Wang**

United States of America

**William Warren**

United States of America

**Kathleen Watson**

United States of America

**Seng Kwee Wee**

Singapore

**Joseph Weir**

United States of America

**Aner Weiss**

Israel

**Jill Whitall**

United States of America

**Maura Whittaker**

Canada

**Jason Wingert**

United States of America

**Hartwig Woldag**

Germany

**Shun-Chi Wu**

Taiwan

**Ming Wu**

United States of America

**Jianhui Wu**

China

**Max Wuehr**

Germany

**Sophie Marie Wurth**

Switzerland

**Hongbo Xie**

Australia

**Feng Xu**

China

**Zhongliang Yang**

China

**Jun Yao**

United States of America

**Aaron Young**

United States of America

**Nuray Yozbatiran**

United States of America

**Bing Yu**

United States of America

**Karl Zabjek**

Canada

**Vladimir Zatsiorsky**

United States of America

**Karl Zelik**

United States of America

**Erika Zemková**

Slovakia

**Joseph Zeni**

United States of America

**Mingming Zhang**

New Zealand

**Qin Zhang**

China

**Xu Zhang**

China

**Yingchun Zhang**

United States of America

**Jun-Tian Zhang**

Canada

